# Activated p53 in the anti-apoptotic milieu of tuberous sclerosis gene mutation induced diseases leads to cell death if thioredoxin reductase is inhibited

**DOI:** 10.1007/s10495-021-01670-4

**Published:** 2021-04-16

**Authors:** ElHusseiny M. M. Abdelwahab, Judit Bovari-Biri, Gabor Smuk, Janos Fillinger, Donald McPhail, Vera P. Krymskaya, Judit E. Pongracz

**Affiliations:** 1 Department of Pharmaceutical Biotechnology, University of Pecs, Pecs, Hungary; 2 Szentagothai Research Centre, University of Pecs, 20 Ifjusag Str., Pecs, 7624 Hungary; 3 Department of Pathology, University of Pecs, Pecs, Hungary; 4 Department of Pathology, Semmelweis University, Budapest, Hungary; 5 National Koranyi Institute of Pulmonology, Budapest, Hungary; 6 Cell Protx Ltd, Aberdeen, UK; 7 Pulmonary, Allergy and Critical Care Division, Department of Medicine, Perelman School of Medicine, University of Pennsylvania, Philadelphia, USA; 8 Department of Pharmaceutical Biotechnology, Faculty of Pharmacy, University of Pecs, 2 Rokus Str, Pecs, 7624 Hungary

**Keywords:** TSC, MTOR, P53, Apoptosis, ROS

## Abstract

Tuberous sclerosis, angiomyolipoma and lymphangioleiomyomatosis are a group of diseases characterized by mutation in tuberous sclerosis genes (TSC 1-2). TSC mutation leads to continuous activation of the mTOR pathway that requires adaptation to increased ATP requirement. With limited treatment options, there is an increasing demand to identify novel therapeutic targets and to understand the correlations between mTOR pathway activation and the lack of cell death in the presence of TSC mutation. In the current study, we demonstrate deregulation of p53 controlled and mitochondria associated cell death processes. The study also reveals that treatment of TSC mutant cells with the drug candidate Proxison combined with reduced concentration of rapamycin can increase production of reactive oxygen species (ROS), can modify miRNA expression pattern associated with p53 regulation and can reduce cell viability.

**Supplementary Information:**

The online version contains supplementary material available at 10.1007/s10495-021-01670-4.

## Introduction

p53 is a widely studied tumour suppressor that controls cell proliferation and caspase dependent apoptosis [1]. Recently, p53 has been linked to non-canonical programmed cell death mechanisms including caspase-independent apoptosis (CIA), ferroptosis, necroptosis (programmed necrotic cell death), autophagy, mitotic catastrophe, paraptosis, pyroptosis, and efferocytosis which process clears dead cells from tissues [1]. Almost all of the above processes are associated with the mitochondrion. In CIA, p53 transcriptionally upregulates apoptosis-inducing factor (AIF) and endonuclease G (EndoG) released from mitochondria [1]. In ferroptosis p53 transcriptionally represses SLC7A11 (solute carrier family member 7; Cystine/glutamate antiporter xCT) leading to accumulation of ROS due to depletion of glutathione biosynthesis, and inhibition of the glutathione-dependent antioxidant enzyme GPX4 (glutathione peroxidase 4) in the mitochondrial TCA cycle (tricarboxylic acid cycle) [1]. In necroptosis p53 transactivates cathepsin Q, indirectly increases the receptor-interacting serine/threonine kinase proteins RIPK1/RIPK3 via the NRF-miR-873 axis and enhances opening of mitochondrial permeability transition pore (PTP) via direct binding of p53 with a PTP regulator cyclophilin D (cypD) [1]. This leads to mitochondrial swelling and induction of necroptosis. During autophagy nuclear p53 induces upregulation of tuberous sclerosis (TSC2) transcription, AMP-activated protein kinase (AMPK), and damage-regulated autophagy modulator (DRAM) levels to promote cell death [2]. Paraptosis can be induced by expression of insulin-like growth factor I receptor (IGF-IR) leading to swelling of the mitochondria or the endoplasmic reticulum (ER) [1].

In a previous study of lymphangioleiomyomatosis (LAM) [3] TSC mutation was associated with morphologically and functionally abnormal mitochondria that did not induce cell death. The process is connected to thioredoxin reductase (TRXR) controlled suppression of ROS production [3].

TSC mutations are particularly disruptive in the phosphoinositide 3 kinase (PI3K)/Protein kinase B (PKB or AKT)/mTOR pathway, as the mTOR pathway is inhibited by the complex of two proteins Hamartin (TSC1) and Tuberin (TSC2). Mutation in the TSC genes allows continuous activation of mTOR resulting in slow growing neoplasms [4]. The mTOR kinase is a master regulator in two complexes. One is the mTORC1, which is formed by mTOR, Raptor, mLST8/GβL, PRAS40, Deptor, and KBP12-rapa. mTORC1 is frequently upregulated in cancer, particularly under increased oncogenic activation of PI3K signalling or inactivation of the lipid phosphatase PTEN [5]. The other complex is mTORC2 formed by mTOR, Rictor, mLST8, DEPTOR, mSin1, and Proctor 1/2, and is phosphorylated directly by AKT at Ser473 for maximal activation [5]. The mTORC2-dependent AKT phosphorylation leads to the activation of mTORC1 leading to indirect suppression of autophagy [5]. Not surprisingly, TSC1 or TSC2 dysfunction causes uncontrolled cell growth [5]. In healthy tissues, activation of mTORC1 and its downstream target ribosomal protein S6 kinase beta-1 (S6K1 or P70S6K) controls interaction between the Mouse double minute 2 homolog (MDM2) and p53. Via the activated S6K1-MDM2 complex p53 induction is promoted. Deactivation of mTOR-S6K1 signalling leads to MDM2 nuclear translocation, which reduces p53 induction and alters p53-dependent cell death. While p53 stimulates MDM2 expression, MDM2 inhibits p53 activity. In response, p53 inhibits mTORC1 through AMPK and REDD1 (regulated in development and DNA damage responses 1) by targeting the TSC1/2 complexes [6]. This auto-regulatory feedback loop guarantees the balance between cell growth, cell cycle arrest and apoptosis. Conversely, in TSC mutation induced diseases the above auto-regulatory loop is shattered leading to constitutive activation of mTORC1 and accumulation of p53. Accumulation of p53 should trigger apoptosis in diseases caused by TSC mutation, yet cells keep on proliferating [7]. Not surprisingly, inhibition of the mTOR pathway and specifically its downstream target S6K1 has become a therapeutic target [8]. mTORC1 is inhibited by rapamycin via inhibition of S6K1 activation [8]. Although the FDA approved rapamycin can slow down disease progression, it cannot offer a cure and has numerous adverse effects that not all patients can tolerate [9]. In such cases discontinuation of treatment is the only solution [9] that leads to rapid disease progression when the only alternative treatment is organ transplantation [10].

Based on the above we theorized that investigation and modulation of p53 and mitochondrial function associated cell death pathways might provide a better understanding of the molecular background of TSC mutation induced diseases and the results might lead to identification of additional targets for therapy.

## Materials and methods

Materials and methods are detailed in the Supplementary material (Supplementary Materials and methods, S. Table 1 and S. Table 2). Briefly, LAM tissue samples were obtained from lung transplant donors for generation of cell lines [11]. Paraffin embedded tissue samples were obtained retrospectively (54034-4/2018/EKU). In the current study, four patient-derived individual LAM cell lines were used: LAM-100, LAM-111C, LAM-D9065 and LAM-HUP. Controls were primary normal, human bronchial smooth muscle cells (BSMC) and normal human lung fibroblasts (NHLF) (Lonza, Basel, Switzerland). The 621-101 cells were derived from an angiomyolipoma (ALM) and carried biallelic inactivation of the TSC2 gene, then 621-103 TSC2+/+ (S103) and 621-102 TSC2−/− (S102) cells were also derived from ALM [12]. Normal, BSMC, NHLF, LAM, S103 and S102 cells were cultured for 3 days using Falcon™ cell culture slides (Thermo Fisher Scientific, Waltham, USA) or cytospins were made from cell suspension and incubated overnight with primary then conjugated secondary antibodies for 1 h. Images were acquired using an Olympus IX-81 (OLYMPUS Corporation, Tokyo, Japan). 5 µm thick primary LAM tissue sections were stained in Mayer’s haematoxylin solution (Sigma-Aldrich, St. Louis, USA) following manufacturer instruction or were analysed by immunohistochemistry for HMB-45 and p53. Histological evaluation was performed with Panoramic MIDI digital slide scanner (3DHistech, Budapest, Hungary). BSMC, NHLF, LAM, S103 and S102 cell cultures were treated with rapamycin and/or Proxison in mono- or in combination treatment (at 10 or 20 nM Rapamycin and 3 µM Proxison, respectively). Western blot was performed immunoreaction was developed with a chemiluminescence HRP substrate and recorded with ImageQuant LAS-4000 imager (GE Healthcare Life Sciences, USA). RNA was isolated with MN NucleoSpin RNA isolation kit (Macherey–Nagel, Düren, Germany). RNA concentration was measured using NanoDrop (Thermo Fisher Scientific, Waltham, USA). Quantitative RT-PCR was performed using SensiFAST SYBR Green reagent (BioLine, London, UK) in an ABI StepOnePlus system (Thermo Fisher Scientific, Waltham, USA). miRNA levels were assessed by Quantstudio 12 k flex miRNA cards (Thermo Fisher Scientific, Waltham, USA). Phospho-Kinases and apoptosis were assessed using Proteome Profiler Human phospho-Kinases (ARY003B) and Apoptosis (ARY009) arrays (R&D Systems, Minneapolis, USA). Images were captured using LAS-4000 (GE Healthcare Bio-Sciences AB Uppsala, Sweden). Cell Viability was assessed using CellTiter-Glo Luminescent Cell Viability Assay Kit (Promega Corp., Madison, WI, USA). Intracellular ROS levels were measured using a green Fluorometric Intracellular ROS Kit (Sigma-Aldrich, St. Louis, USA). Thioredoxin reductase (TrxR) activity was measured using a thioredoxin reductase assay kit (Abcam, MA, USA, ab83463). Apoptosis was assessed using Annexin V-PE and 7-AAD (BioLegend, San Diego, CA, USA). Statistical analysis was performed using the SPSS program. The S102 and S103 data are presented as mean ± technical error using the student t-test. Data of primary LAM samples and their controls (BSMC n = 4 and NHLF n = 4) are presented as mean ± standard error using one-way ANOVA. p < 0.05 was considered as significant.

## Results

### TSC mutation is associated with molecular changes affecting mitochondrial function and apoptosis

**Fig. 1:**
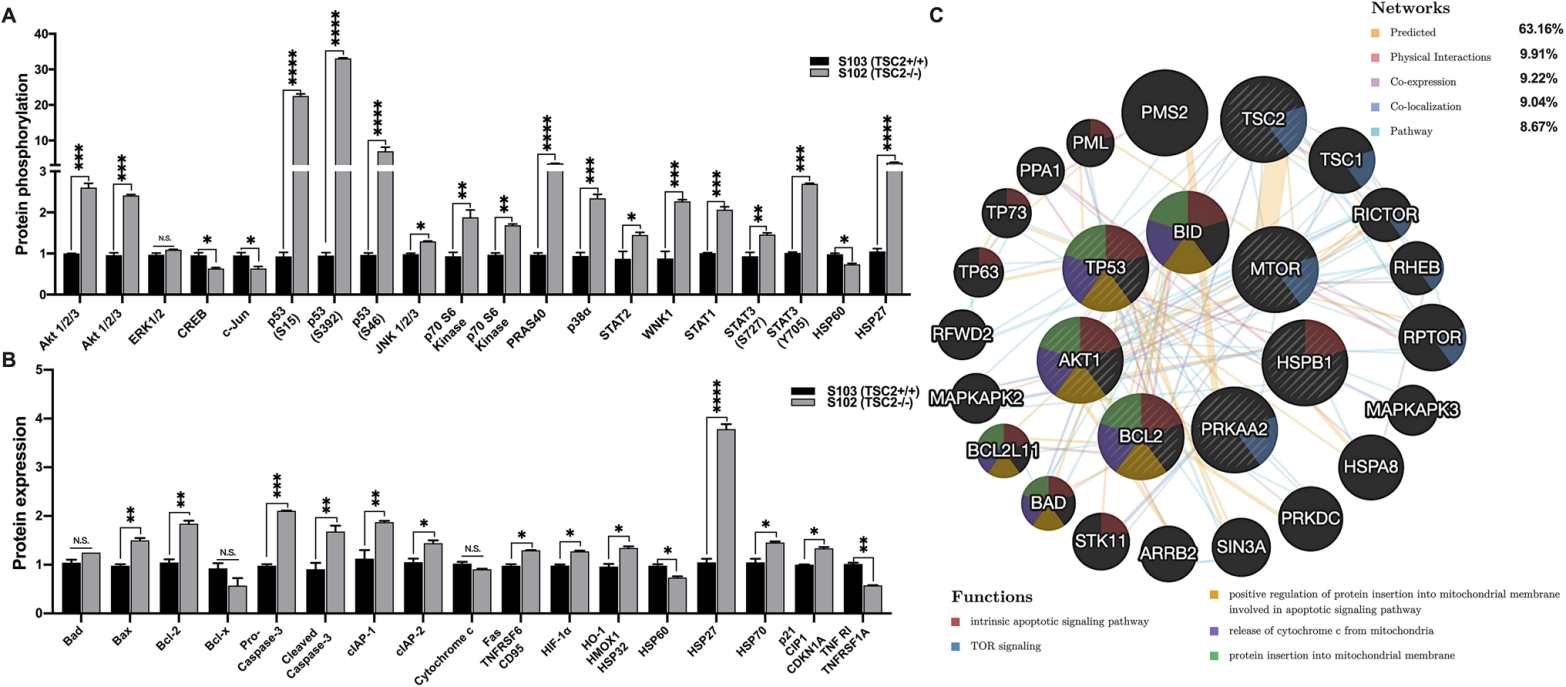
Constitutive activation of the mTOR pathway due to TSC mutation deregulates apoptosis associated protein expression and phosphorylation. **a** Protein phosphorylation using Phospho-Kinase protein array of S102 compared to S103 cell lines (n = 3). Significant changes are marked as *, **, *** and **** (p < 0.05, p < 0.001, p < 0.0002 and p < 0.0001, respectively). **b** Apoptosis associated protein expression levels using apoptosis protein arrays of S102 compared to S103 cell lines (n = 3). Significant changes are marked as *, **, *** and **** (p < 0.05, p < 0.001, p < 0.0002 and p < 0.0001, respectively). **c** Protein–protein interactions using linear a regression-based prediction algorithm analysis GeneMANIA

mTOR is downstream of the PI3K and AKT enzymes that transduce signals from growth factor activated receptor kinases [4]. To investigate kinase activation and their effects on apoptosis, protein expression and protein phosphorylation analysis was performed in TSC mutant ALM cell lines S102(TSC2−/−) and S103(TSC2+/+) [12] using Proteome Profiler Human Phospho-Kinase and Apoptosis Arrays (Fig. 1a, b). Out of the 43 kinases phosphorylation of 20 were significantly deregulated (Fig. 1a). Similarly, the apoptosis arrays revealed a general protein expression deregulation of both pro- and anti-apoptotic factors (Fig. 1b). The kinases with the most increased phosphorylation levels included AKT and its target PRAS40; c-Jun N-terminal kinases (JNK) 1, 2 and 3, P70S6, the lysine deficient protein kinase 1 (WNK1) and p38alpha. Phosphorylation of cAMP-response element binding protein (CREB), transcription factor c-Jun and heat shock protein 60 (HSP60) were reduced. In parallel, phosphorylation of the signal transducer and activator of gene transcription (STAT1, 2, 3) (S727, S705), heat shock protein 27 (HSP27) were significantly increased (Fig. 1b). Activity of the tumour suppressor and apoptosis inducer p53 has a highly complex regulation [13, 14] and its actual function depends on post-translational modifications including variable phosphorylation at the 24 phosphorylation sites [15]. In the study p53 was phosphorylated at three important serine phosphorylation cites S15, S392 and S46 (Fig. 1b), still the TSC2−/− cell line proliferated faster than the TSC2+/+ control (S. Fig. 1).

To understand the significance of the results, a linear regression-based prediction algorithm analysis [16] was used to predict protein–protein interactions amongst expression and phosphorylation of kinases and apoptosis regulating proteins (Fig. 1c, S. Table 3). The analysis revealed that several proteins are not only members of the same pathway, regulate apoptosis as well as mitochondrial function, but often physically co-localize (Fig. 1c).

**Fig. 2:**
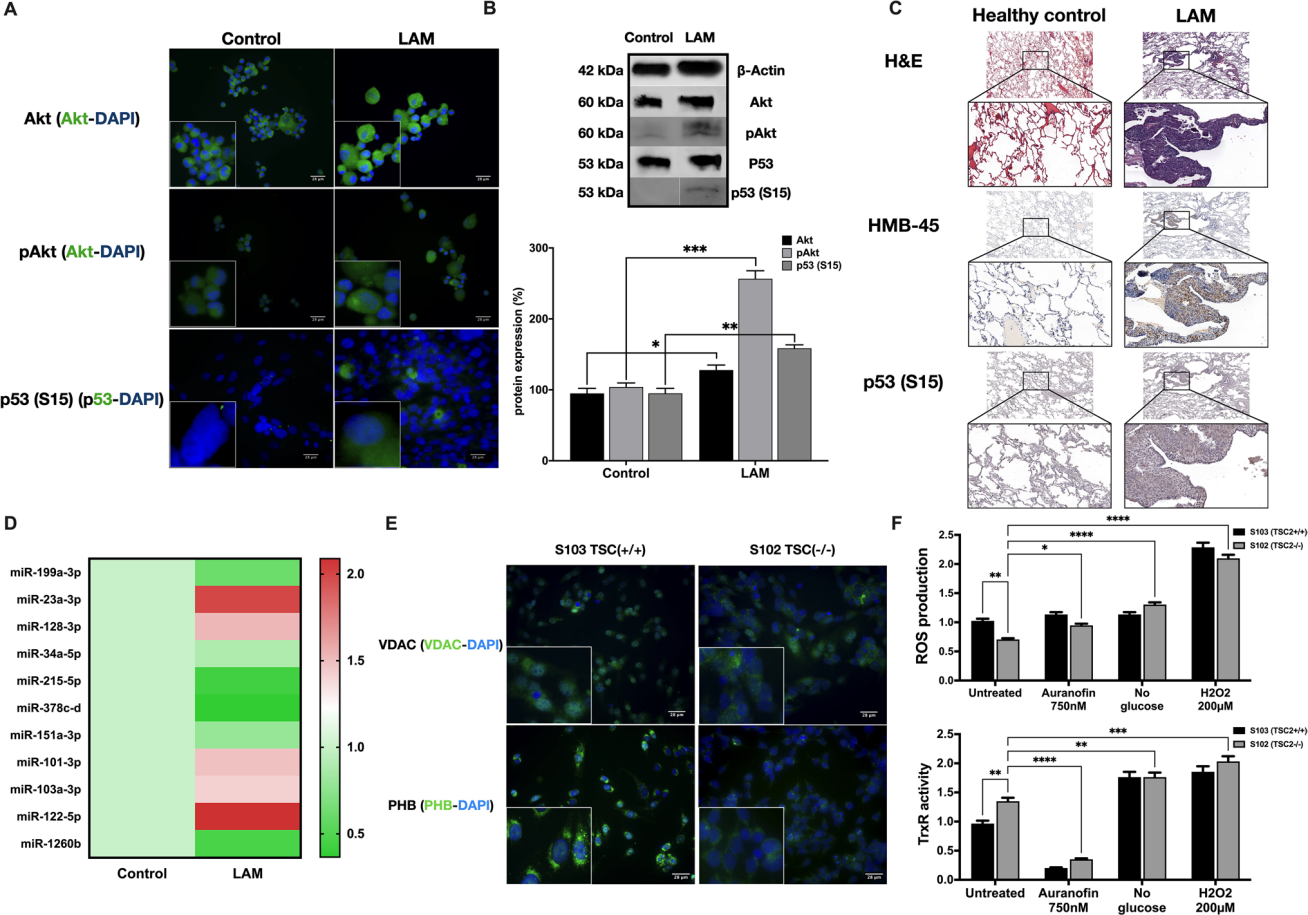
Apoptosis and mithochondria associated molecular expression and phosphorylation. **a** P-AKT and p53 (S15) immunofluorescent staining (magnification × 40, size bar 28 μm). **b** p-AKT WB of LAM primary cell lines (pooled samples n = 4) and control cell lines (n = 4). p53 LAM primary cell lines (n = 4). Data is presented as % of protein expression (n = 3). **c** H&E, HMB-45 (clinical diagnostic LAM marker) and p53 immunohistochemistry of primary LAM lung sections (n = 6) and healthy controls (n = 3). **d** miRNA expression involved in regulation of apoptosis, p53 activation and proliferation. **e** S102 and S103 cell lines IF staining for PHB and VDAC1 (magnification × 40, size bar 28 μm). **f** ROS production (fluorescence intensity) and TRXR activity (nmol/min/ml) (n = 3). Significant changes are marked as *, **, *** and **** (P < 0.05, P < 0.001, P < 0.0002 and P < 0.0001 respectively)

**Fig. 3:**
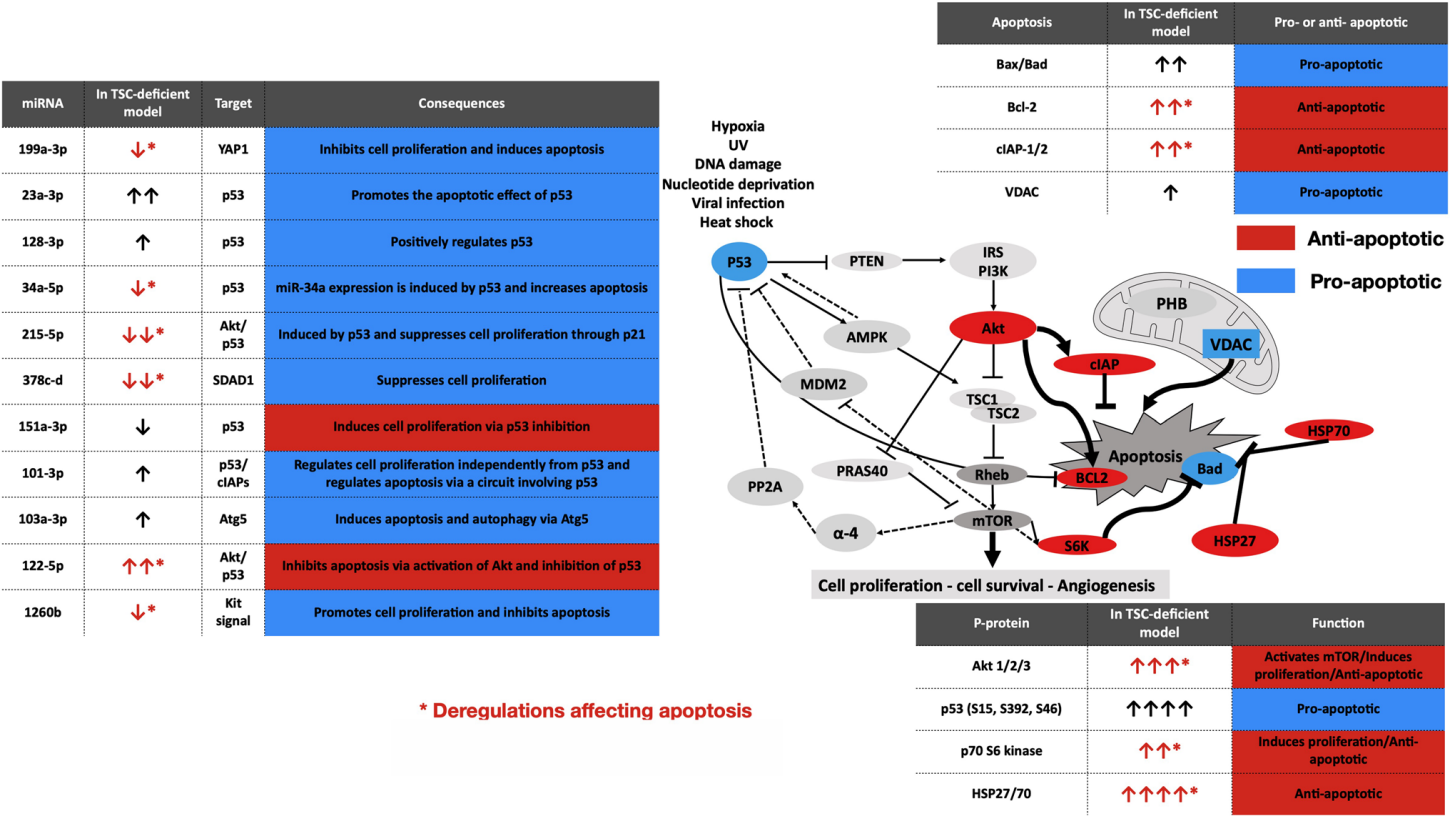
Connections amongst kinases, mitochondria, apoptosis and regulatory miRNA. Deregulations affecting apoptosis are marked by a red asterisk (*) (Color figure online)

To confirm that deregulation of the pro- and anti-apoptotic pathways are not a unique feature of the AML derived S102 cell line, 4 primary LAM tissue derived cell lines (Fig. 2a, b, S. Fig. 2) and primary LAM lung tissue sections (Fig. 2c) were analysed. IF, WB and IHC all showed parallel upregulation of p53 (S15) and AKT phosphorylation (Fig. 2a–c). As protein expression is also regulated by miRNA-s, several miRNA-s that are known to control the apoptotic process were also tested in TSC2−/− LAM cell lines and were compared to TSC2+/+ primary BSMC-s (Fig. 2d). Several miRNA-s directly or indirectly activating the AKT, PI3K/AKT/mTOR pathway were deregulated and their effects are summarised in Fig. 3 and S. Table 4. Specific miRNAs and pro-apoptotic members of the Bcl-2 family (e.g. Bax) (Fig. 1a) regulate the opening of the mitochondrial voltage-dependent anion channel (VDAC) [17, 18]. Opening of VDAC leads to loss in membrane potential and consequent release of cytc in ferroptosis [1]. As VDAC binds Prohibitin (PHB), an inner mitophagy receptor that is an essential protein for mitochondrial integrity and cristae morphology [19], both protein levels were visualized by fluorescent staining. While expression of VDAC was increased, PHB levels were reduced in TSC2−/− S102 compared with TSC+/+ S103 controls (Fig. 2e). As to demonstrate the power struggle between pro- and anti-apoptotic pathways, ROS production was also tested. Although ROS is a normal product of cellular metabolism [20], mitochondrial damage can lead to increased accumulation of ROS and cell death. However, cells can be protected from ROS by a cellular antioxidant defence system involving thioredoxin reductase (TRXR) and glutathione reductase [21]. While TRXR activity was increased, ROS production was reduced in TSC2−/− cells compared to TSC2+/+ controls (Fig. 2f). To test whether inhibition of TRXR can lead to increased ROS production, both the mutant and control cell lines were treated with 750 nM Auranofin, a well-known inhibitor of TRXR. Treatment with Auranofin significantly reduced TRXR activity and increased ROS levels indicating that the anomaly in ROS production is associated with increased TRXR enzyme activity in the TSC mutant cell lines (Fig. 2f, S. Fig. 3).

Based on the data, it seemed that although, p53 expression and phosphorylation is significantly increased in TSC2 mutant cells (Figs. 1, 2), activation of p53 is not sufficient to overcome the anti-apoptotic milieu (Fig. 3).

### Reduced concentration of rapamycin in combination with drug candidate Proxison increases ROS production and apoptosis

**Fig. 4:**
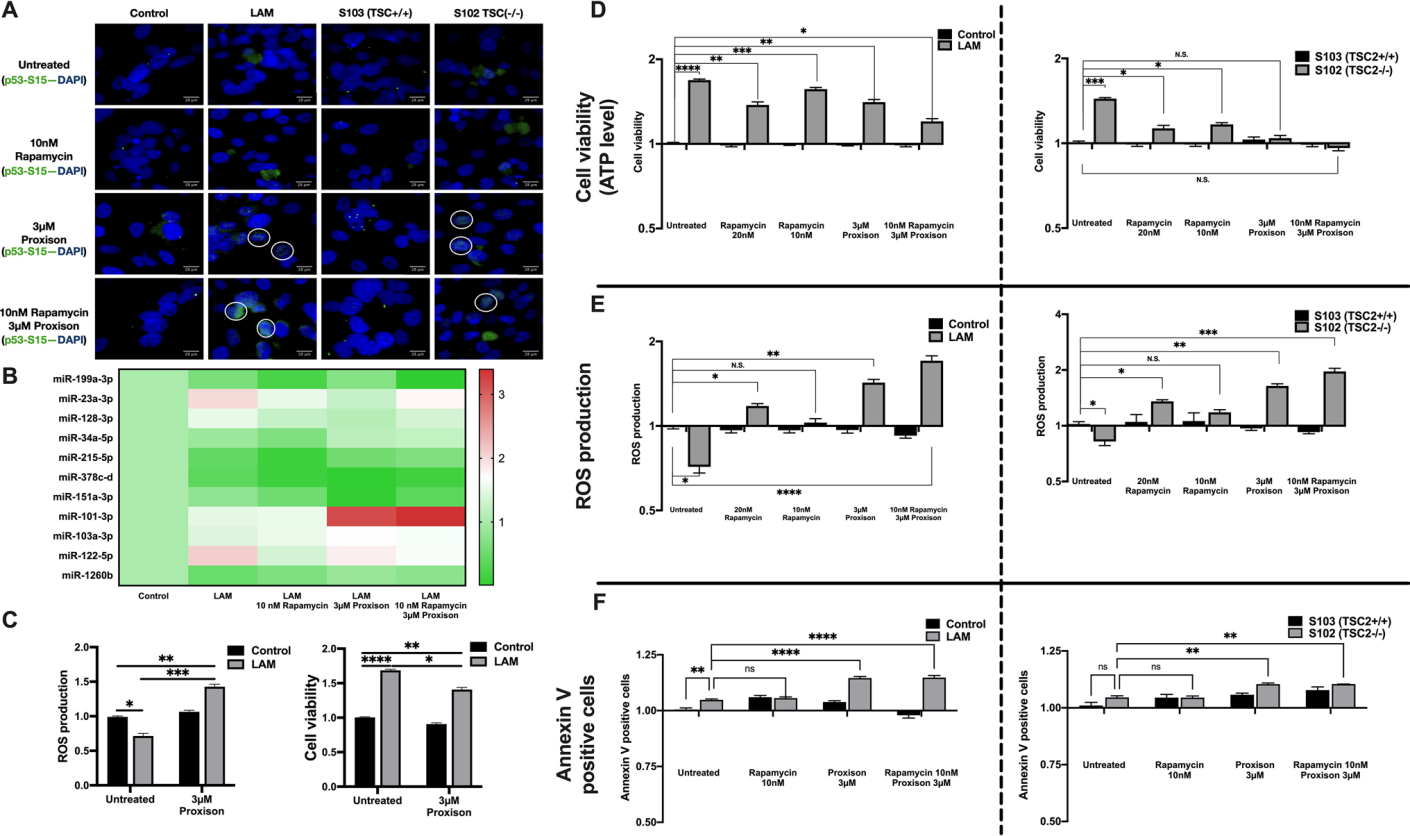
Effects of Proxison (3 μM) and/or rapamycin (10 and 20 nM) on p53, mi-RNA expression, ROS production and cell viability in primary LAM lung and AML derived cell lines. **a** p53 localization detected by immunofluorescent staining (magnification × 40, size bar 28 μm) (n = 3). White circle indicates nuclei with p53. **b** Apoptosis regulating miRNA levels following treatment, **c** ROS and ATP levels (cell viability) of LAM cells after 3 μM Proxison treatment, **d–f** Comparative analysis of ATP, ROS and Annexin V positivity in S102 and LAM cells compared to their respective controls (n = 3). Significant changes are marked as *, **, *** and **** (P < 0.05, P < 0.001, P < 0.0002 and P < 0.0001, respectively)

Loss of TSC2, the negative regulator of mTORC1 results in accumulation of p53 that ought to trigger apoptosis. In contrast, TSC-deficient cells proliferate and resist apoptosis due to complex alteration of the expression and activation network of pro- and anti-apoptotic proteins, regulatory miRNAs involved in apoptosis (Fig. 3). To investigate if the complex deregulation of the apoptotic mechanisms could be restored, the FDA approved rapamycin and a flavonoid-based drug candidate, Proxison were tested [22]. In our earlier study, Proxison restored mitochondrial morphology and normalized mitochondrial function [3]. In the current test TSC mutant cell lines and their controls were exposed to 3 µM Proxison and/or standard (20 nM) or reduced concentration (10 nM) of rapamycin. Fluorescent staining of the cell lines has shown increased nuclear localization of p53 (S15) after 3 µM Proxison as well as 10 nM rapamycin and 3 µM Proxison combination treatment (Fig. 4a, S. Fig. 4) [23]. Expression levels of miRNAs were mostly normalised while the anti-proliferation and anti-invasion miR-101-3p was significantly upregulated after treatment with 3 µM Proxison both in mono- or in combination treatment with 10 nM rapamycin (Fig. 4b). Cell viability was assessed by quantifying cellular ATP levels, ROS production and Annexin V levels after 20 nM or 10 nM rapamycin and/or 3 µM Proxison treatment. 3 µM Proxison significantly increased ROS production in all TSC mutant cell lines, while decreased ATP levels and increased Annexin V (Fig. 4d–f). Although 20 nM rapamycin significantly increased ROS levels, 10 nM rapamycin did not differ from the control. Combination of rapamycin at 10 nM and 3 µM Proxison had similar effects to Proxison mono-treatment but significantly increased ROS production and reduced ATP levels keeping Annexin V at the same increased level as it was detected after Proxison mono-treatment (Fig. 4d–f, S. Figure 5 and 6).

## Discussion

The mTOR pathway plays a crucial role in regulating cellular proliferation, mitochondrial biogenesis, cellular metabolism and apoptosis [24].

In the present study we focused our attention on malfunction of apoptotic pathways as a result of constitutive activation of mTOR. The significantly increased expression and phosphorylation of p53 (S15 and 46) in TSC2 mutant cell lines revealed a distortion in the pro- and anti-apoptotic balance. The cell death processes are blocked in TSC mutant cells despite p53 activation (Summarized in Fig. 3) largely due to the dysfunctional feedback circuit between mTOR and p53 [2]. The AKT-AMPK-mTOR-P70S6K pathway plays a crucial role in the regulation of p53 activity [2]. Phosphorylation of the AKT target PRAS40 an inhibitor of the mTORC1 complex [8] was increased that can lead to dissociation from Raptor and resulting in activation of mTORC signalling [8]. mTORC1 activation inhibits PP2A, enabling the accumulation of the oncogenic protein cMyc that contributes to the proliferation-promoting effects of the PI3K/AKT/Myc pathway [25, 26]. Additionally, PRAS40 can bind p65, a subunit of the canonical NF-κB transcription factor heterodimer consequently modulating NF-κB transcriptional activity that controls cell growth, inflammation and stress responses [8]. In parallel, upregulation of expression and increased phosphorylation of the cytoprotective, anti-apoptotic HSP27 can lead to the activation of TAK1 and TAK1-p38/ERK pro-survival signalling that opposes tumour necrosis factor (TNF)-alpha-induced apoptosis [27, 28]. HSP27 can also enhance the activation of the NF-κB pathway [27]. Additionally, HSP27 can inhibit ROS production via raising glutathione levels [25]. While the pro-apoptotic TRAIL is downregulated, no changes were detected in cytochrome c (cytc), a small electron transport haemoprotein loosely associated with the inner membrane of the mitochondrion [17] and characteristically released from the mitochondria during CIA [17]. This is particularly important, as HSP27 negatively interferes with apoptosis by inhibiting cytc-mediated activation of caspases in the cytosol [17]. When cytc is released from the mitochondria and HSP27 binds to cytc then cytc-mediated interaction of Apaf-1 with pro-caspase-9 is prevented [27]. Additionally, many of the Bcl-2 family members reside in the outer mitochondrial membrane, oriented towards the cytosol. The cytoprotective Bcl-2 family proteins such as Bcl-2 and Bcl-XL prevent mitochondrial permeability and the release of pro-apoptotic proteins. The expression of p53 regulated VDAC was increased and PHB reduced affecting ferroptosis. Simultaneously, the anti-apoptotic factor of Bcl-2 and the pro-apoptotic Bax were significantly upregulated when increased HSP27 phosphorylation is detected [25]. Furthermore, the cellular inhibitor of Apoptosis Protein 1 and 2 (cIAP 1, 2) that directly inhibit caspases were significantly up-regulated together with STAT 1, 2 and 3 that interfere with mitochondrial activity and protein homeostasis. The inhibitory signals associated with p53 are relayed to mTOR via the malfunctioning AMPK-TSC pathway which breaks the feedback circle if TSC genes are mutated [6, 29].

We have also revealed that TSC mutation leads to deregulation of miRNA levels that regulate cellular proliferation, p53 expression and phosphorylation as well as apoptosis. We also consistently detected upregulation of anti-ROS reductive mechanisms disallowing mitophagy and autophagy in the presence of TSC mutation.

Based on the accumulated knowledge about p53, increased amount and phosphorylation at sites of Serine 15, 20 and/or 46 should promote transactivation of pro-apoptotic target genes [30].

While mere phosphorylation of p53 is not enough to induce any of the studied pathways to trigger apoptosis, targeting deregulated phosphorylation cascades, mitochondrial function and metabolic enzyme levels can potentially normalize cell death mechanisms. Proxison, that targets mitochondria associated gene expression, decreases TRXR activity [3], enhances ROS production [20] and reduces cell viability presents a controversy. As Proxison can inhibit TRXR, detection of increased ROS levels is not surprising. However, Proxison is flavonoid based consequently; it can also reduce ROS levels. Therefore it appears that the function of Proxison depends on the balance its pro- and anti-apoptotic activity. Interestingly, 20 nM rapamycin (in vitro concentration roughly equivalent to therapeutic dosage) failed to reduce cell viability. Reduced concentration of rapamycin (10 nM) in combination with 3 µM Proxison induced apoptosis more efficiently than either drug or drug candidate in monotreatment. Application of reduced rapamycin dosage would have clinical significance as it could reduce rapamycin-induced side effects in patients.

## Conclusion

p53 is the centre of various cellular signalling pathways. p53 is regulated post-translationally and affected by an excess number of enzymes resulting in a dualistic role of p53 in pro- and anti-apoptotic mechanisms. Therefore, it is challenging to find the correct targets to modulate p53-associated signalling pathways to the desired direction. Identification of the parallel regulatory mechanisms can help to identify therapeutic targets in TSC mutation triggered diseases. Currently, the strong anti-apoptotic microenvironment in cells with TSC mutation cannot be completely overcome by the FDA approved drug, rapamycin. Our study shows that lower dosage of rapamycin (10 nM) combined with the drug candidate Proxison (3 µM) can result in cell death. Although further studies are required, combined inhibition of the mTOR pathway and TRXR can potentially lead to a more effective therapy.

## Supplementary Information

Below is the link to the electronic supplementary material.

Supplementary file1 (DOCX 7450 kb)

## Data Availability

All data are available from the corresponding author upon reasonable request.
