# Supplementary material for: Activated p53 in the anti-apoptotic milieu of tuberous sclerosis gene mutation induced diseases leads to cell death if thioredoxin reductase is inhibited
Source: Apoptosis. 2021 Apr 16;26(5-6):253–60. doi: 10.1007/s10495-021-01670-4 (PMC8197715; doi:10.1007/s10495-021-01670-4)
Supplement: Supplementary file 1 — Supplementary file1 (DOCX 7450 kb) [file 10495_2021_1670_MOESM1_ESM.docx]

**Supplementary material and data - Highlighted**

**Materials and methods**

LAM tissue samples were obtained from lung transplant donors for generation of cell lines, in accordance with the Declaration of Helsinki, approved by the Institutional Review Board at the University of Pennsylvania [1] and provided by the National Disease Research Interchange (NDRI, Philadelphia, PA). LAM patients had given written informed consent and all the collected samples were treated anonymously. Paraffin embedded tissue samples were obtained retrospectively from the Departments of Pathology at Semmelweis University, Budapest, and from the University of Pecs, Pecs, Hungary and the National Koranyi Institute of Pulmonology, Budapest, Hungary. The study was approved by the Medical Research Council of Hungary (54034-4/2018/EKU).

## LAM cell lines, bronchial smooth muscle cells (BSMC), normal human lung fibroblast (NHLF) S102 and S103 cell lines and cell culture conditions

Primary cultures of human LAM cells where established in the Department of Medicine, University of Pennsylvania, Pennsylvania, USA [1]. Briefly, the primary cultures of LAM cells were dissociated from the LAM nodules of transplant patients. Each LAM nodule was used to establish individual cell lines (characterized based on alpha smooth muscle actin (α***-***SMA) expression, mTORC1 activation, HMB45 immunoreactivity, DNA synthesis, and cell migration) [2]. In the current study, four patient-derived individual LAM cell lines were used including LAM-100, LAM-111C, LAM-D9065 and LAM-HUP. As controls, primary cultures of normal, human bronchial smooth muscle cells (BSMC) and normal human lung fibroblasts (NHLF), were purchased from Lonza (Basel, Switzerland). Normal, BSMC and LAM cells were cultured at 37°C, 5% CO_2_ in SMC Growth Medium (insulin, hFGF, GA, FBS and hEGF) (Lonza, Basel, Switzerland). The 621-101 cells were derived from an angiomyolipoma and carried bi-allelic inactivation of the TSC2 gene [3, 4]. The 621-102 cell line was generated by introduction of E6/E7 (pLXSN 16E6E7-neo) and human telomerase (pLXSN hTERT-hyg) into a primary culture of TSC2 null human angiomyolipoma cells [5]. 621-103 was generated by stable transfection of TRI102 with wild-type TSC2 (pcDNA3.1 TSC2-zeo) into 621-101 cells [3].

## Haematoxylin eosin staining

5 µm thick tissue sections were stained in Mayer’s haematoxylin solution (Sigma-Aldrich, St. Louis, USA) for 10 min, washed, then differentiated with 0.25% acetic acid and in eosin solution. Sections were mounted using Vectashield mounting medium (Vector Laboratories, Burlingame, USA). Images were taken using Nikon Eclipse Ti-U inverted microscope.

## Immunofluorescent staining

Normal, BSMC, NHLF, LAM, S103 and S102 cells were cultured for 3 days using Falcon™ chambered cell culture slides (Thermo Fisher Scientific, Waltham, USA) or Cytospins were made from cell suspension. Cell cultures were then fixed in 4% formaldehyde and permeabilized using PBS containing 0.1% Triton-X and 5% BSA.

**S. Table 1.** Antibodies used in immunofluorescent staining, immunohistochemistry and western blotting

| Anti-alpha -Smooth Muscle Actin | MAB1420 (1∶100) |
| --- | --- |
| Anti-mTOR Antibody | ab25880 (1:100) |
| Anti-p70 S6 kinase Antibody | Ab32529 (1:100) |
| anti-HMB-45 | Ab787 (1:100) |
| Anti-human Akt | Cell Signaling 9272 (1:1000) |
| Anti-human pAkt | Cell Signaling 9275 (1:0000) |
| Anti-human P53 | ab1431 (1:200) |
| Anti-beta actin | Cell Signaling 4970 (1:0000) |
| Anti-mouse Alexa 488 | A28175 (1:200) |
| Anti-rabbit Alexa 647 | A27040 (1:200) |
| Anti-rabbit Alexa 488 | A11034 (1:200) |
| Anti-mouse Alexa 647 | A32728 (1:200) |
| Anti-mouse HRP | 170-6516 (1:2000) |
| Anti-rabbit HRP | 170-6515 (1:2000) |
| Anti-rabbit HRP | P0448 (1:200) |

Nuclei were counter stained with DAPI. Images were acquired using an Olympus IX-81 (OLYMPUS Corporation, Tokyo, Japan) both light and fluorescence microscope.

**Immunohistochemistry**

5 µm thick tissue sections were stained using immunohistochemistry. First, the slides were rinsed in heated xylene and were washed with a descending series of alcohol to remove paraffin. After deparaffination the slides were rehydrated in distilled water and antigen retrieval was performed by heating the slides in Target Retrieval Solution (pH 6, DAKO, Produktionsvej, Denmark) at 97°C for 20–30 min. Subsequently slides were washed in dH_2_O and endogenous peroxidase activity was blocked with 3% H_2_O_2_ containing TBS (pH 7.4) for 15 min. Then slides were washed three times with TBS containing Tween (0.05%, pH 7.4). Pre-blocking was carried out with 3% BSA in TBS for 20 min before overnight incubation with anti- Melanoma gp100 antibody (HMB-45) (1:100, HMB-45 mouse monoclonal antibody clone: Ab787, ABcam) primary antibody at 4°C. Following incubation slides were washed with TBS for three times then incubated with peroxidase conjugated secondary antibody (1:100, Polyclonal Goat Anti-Rabbit IgG, DAKO) for 90 min. Antibody labelling was visualized with the help of liquid DAB Substrate Chromogen System (DAKO). For nuclear counterstaining, haematoxylin staining was performed. Finally, slides were mounted with Faramount Aqueous Mounting Medium (DAKO, Produktionsvej, Denmark). Histological evaluation was performed with the help of Panoramic MIDI digital slide scanner (3DHistech, Budapest, Hungary). Image analysis was performed using ImageJ software with IHC toolbox plug-in.

### **Rapamycin, Proxison, Auranofin and H_2_O_2_ treatment**

BSMC, NHLF, LAM, S103 and S102 cell cultures were treated with mono rapamycin or Proxison treatments and in combined treatments using the following concentrations: 10 or 20 nM Rapamycin catalogue: tlrl-rap (InvivoGen, San Diego, USA) and 3 µM Proxison (Antoxis Ltd, Aberdeen, UK) for 24h at 37°C, 5% CO_2_. Auranofin and H_2_O_2_ were used as positive controls for TrxR activity, ROS production and apoptosis analysis (Annexin V) using the following concentrations: 750 nM Auranofin (Thermo Scientific, Waltham, MA) and 200 µM H_2_O_2_ (Thermo Scientific, Waltham, MA).

**Western blot**

Cells were lysed in ice-cold RIPA buffer (Sigma-Aldrich, St. Louis, USA) supplemented with protease inhibitors (Roche Diagnostics, Mannheim, Germany) for 30 min on ice and centrifuged at 16,000 × g for 20 min at 4° C. The supernatant was then used as the cell lysate. The protein content of each cell lysate was assessed using a Qubit protein assay kit (Thermo Scientific, Waltham, MA). 30 µg of total protein was loaded onto Mini Protean gel (Bio-Rad, California, USA), then electrophoresis was followed by overnight blotting onto a nitrocellulose membrane using 10 mA current. The blots then were blocked in 5% non-fat skimmed milk blocking solution (Bio-Rad, California, USA) in TBS-T for 1 h and incubated with primary antibodies diluted 1:1000 in 2.5% non-fat skimmed milk powder in TBS-T overnight at 4° C. After washing with TBS-T, the blots were incubated with rabbit anti-goat/HRP diluted in 2.5% non-fat skimmed milk powder in TBS-T for 1 h at room temperature. The immunoreaction was developed with a chemiluminescence HRP substrate and recorded with ImageQuant LAS-4000 imager (GE Healthcare Life Sciences, USA).

## RNA isolation

Total RNA was extracted from normal BSMC, NHLF and LAM cell cultures with MN NucleoSpin RNA isolation kit according to the manufacturer’s protocol (Macherey-Nagel, Düren, Germany). The concentration of RNA samples was measured using NanoDrop (Thermo Fisher Scientific, Waltham, USA). Total RNA from human lung tissues were obtained using TRIzol reagent (Invitrogen, Thermo Fisher Scientific, Waltham, USA). RNA (1 µg) was digested with DNase (Sigma-Aldrich, St. Louis, USA) to eliminate any DNA contamination. cDNA was synthesized with high capacity RNA to cDNA kit (Thermo Fisher Scientific, Waltham, USA). Reverse transcription was performed with random hexamer primers.

## Quantitative qRT-PCR

qRT-PCR was performed using SensiFAST SYBR Green reagent (BioLine, London, UK) in an ABI StepOnePlus system. Gene expressions using sequence specific primers (Table 2) were analysed with StepOne software and normalized to beta-actin. Changes in gene expression were calculated according to the 2^-ddCt^ method.

**S. Table 2.** Primer sequences

| **Gene name** | **Forward primer** | **Reverse primer** |
| --- | --- | --- |
| **β-actin** | GCGCGGCTACAGCTTCA | CTTAATGTCACGCACGATTTCC |
| **TSC1** | CCGTGGCCCTATGCTTGTAA | CGGCTTTGCCCACATATTCG |
| **TSC2** | CCTTGGACGGTATTGCCTGT | GCCTGCTTCTGTGTACCACT |

**Quantstudio 12k flex**

cDNA was prepared using TaqMan miRNA reverse transcriptase kit and Megaplex RT primers Pool A and B (Thermo Fisher Scientific, Waltham, USA) according to manufacturers’ protocol using 350ng-1000ng of total RNA as starting material. miRNA expression levels were performed using open array miRNA card Pool A and B and Quantstudio 12k flex (Thermo Fisher Scientific, Waltham, USA).

**Protein arrays**

Cell lysates of 1 x 107 103 cells/ml were assessed using a Human phosphor-kinases and apoptosis Array Kits (R&D Systems, Minneapolis, USA). Protein concentration was determined using a fluorescent protein assay (Qubit Protein, Thermo Fisher Scientific, Waltham, USA). Briefly, the Detection Antibody Cocktail was mixed with each sample and incubated with the membrane at 4°C overnight, then with Streptavidin-HRP at room temperature finally with the Chemiluminescent-Reagent Mix. Images were captured using LAS-4000 (GE Healthcare Bio-Sciences AB Uppsala, Sweden), and intensity was determined using ImageJ (https://imagej.nih.gov/ij/) and normalized to the reference spots.

**Cell Viability Assay**

CellTiter-Glo Luminescent Cell Viability Assay Kit (Promega Corp., Madison, WI, USA) was used to evaluate cell viability after drug treatment. Cells were seeded into 96-well plates, after 24 h incubation and were treated with mono rapamycin or RA or Proxison and their combinations. After incubation for 24 h at 37°C, 100 μl of CellTiter-Glo reagent were added and luminescence measured with EnSpire® Multimode Plate Reader (PerkinElmer, Waltham, Massachusetts, USA). Pierce™ BCA Protein Assay Kit (Thermo Fisher Scientific, Waltham, USA) was used to measure protein content and results are presented as the fold change vs. control.

**Measurement of intracellular ROS levels**

Reactive oxygen species were measured using green Fluorometric Intracellular Ros Kit (Sigma-Aldrich, St. Louis, USA). Cells were grown in 96-well plates and treated then ROS levels were assessed using a detection kit following the manufacturer’s instructions and results were measure at Ex/Em = 490/525 nm with EnSpire® Multimode Plate Reader (PerkinElmer, Waltham, Massachusetts, USA). Pierce™ BCA Protein Assay Kit (Thermo Fisher Scientific, Waltham, USA) was used and results are presented as the fold change vs. control.

**Measurement of Thioredoxin Reductase Activity**

Thioredoxin reductase (TrxR) activity was measured in cell homogenates using a thioredoxin reductase assay kit according to the manufacturer's instructions (Abcam, MA, USA, ab83463). Briefly, cells were cultured in 96-well plates and incubated with treatment for 24 h at 37°C. Cells were collected and homogenized on ice by adding 100 μL cold assay buffer. Supernatants were collected by centrifugation at 10,000 ×g for 15 min, and TrxR activity was measured according to the kit's instructions.

**Flow cytometry**

50,000 cells were collected from each cell cultures and incubated with Annexin V-PE and 7-AAD (BioLegend, San Diego, CA, USA) for 15 mins at 37^o^C. Using BD FACSCanto™ II system (three lasers: blue 488-nm, air-cooled, 20-mW solid state, red 633-nm, 17-mW HeNe, and violet 405-nm, 30-mW solid state). Labeled cells were analyzed using FACS Canto II flow cytometer (BD Immunocytometry Systems, Erembodegen, Belgium) with BD FACS DIVA software V6 and data were analyzed by FCS Express V3 software.

## Statistical analysis

Unless otherwise noted, statistical analysis was performed with SPSS version 20 software. S102 and S103 data are presented as mean ± technical error of three replicates and statistical analysis was performed using the t-test. LAM primary samples and their controls (BSMC n=4 and NHLF n=4) data are presented as mean ± standard error of mean (SEM), and statistical analysis was performed using the one-way ANOVA. p<0.05 was considered as significant.

1. Goncharova EA, Goncharov DA, Eszterhas A, et al (2002) Tuberin regulates p70 S6 kinase activation and ribosomal protein S6 phosphorylation: A role for the TSC2 tumor suppressor gene in pulmonary lymphangioleiomyomatosis (LAM). J Biol Chem 277:30958–30967. https://doi.org/10.1074/jbc.M202678200

2. Goncharova EA, Goncharov DA, Lim PN, et al (2006) Modulation of cell migration and invasiveness by tumor suppressor TSC2 in lymphangioleiomyomatosis. Am J Respir Cell Mol Biol 34:473–480. https://doi.org/10.1165/rcmb.2005-0374OC

3. Yu J, Astrinidis A, Howard S, Henske EP (2004) Estradiol and tamoxifen stimulate LAM-associated angiomyolipoma cell growth and activate both genomic and nongenomic signaling pathways. Am. J. Physiol. - Lung Cell. Mol. Physiol. 286

4. Carsillo T, Astrinidis A, Henske EP (2000) Mutations in the tuberous sclerosis complex gene TSC2 are a cause of sporadic pulmonary lymphangioleiomyomatosis. Proc Natl Acad Sci U S A 97:6085–6090. https://doi.org/10.1073/pnas.97.11.6085

5. Furukawa T, Duguid WP, Rosenberg L, et al (1996) Long-term culture and immortalization of epithelial cells from normal adult human pancreatic ducts transfected by the E6E7 gene of human papilloma virus 16. Am J Pathol 148:1763–1770

S. Table 3. GeneMANIA gene expression analysis.

*
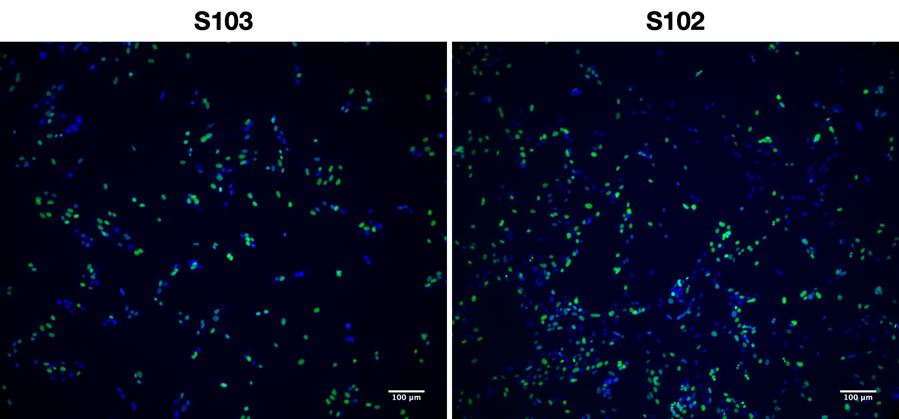
*

**S. Figure 1. Proliferation capacity of S102 compared to S103 using BrdU assay (BrdU green, DAPI blue, size-bar 100 μm)**.

**
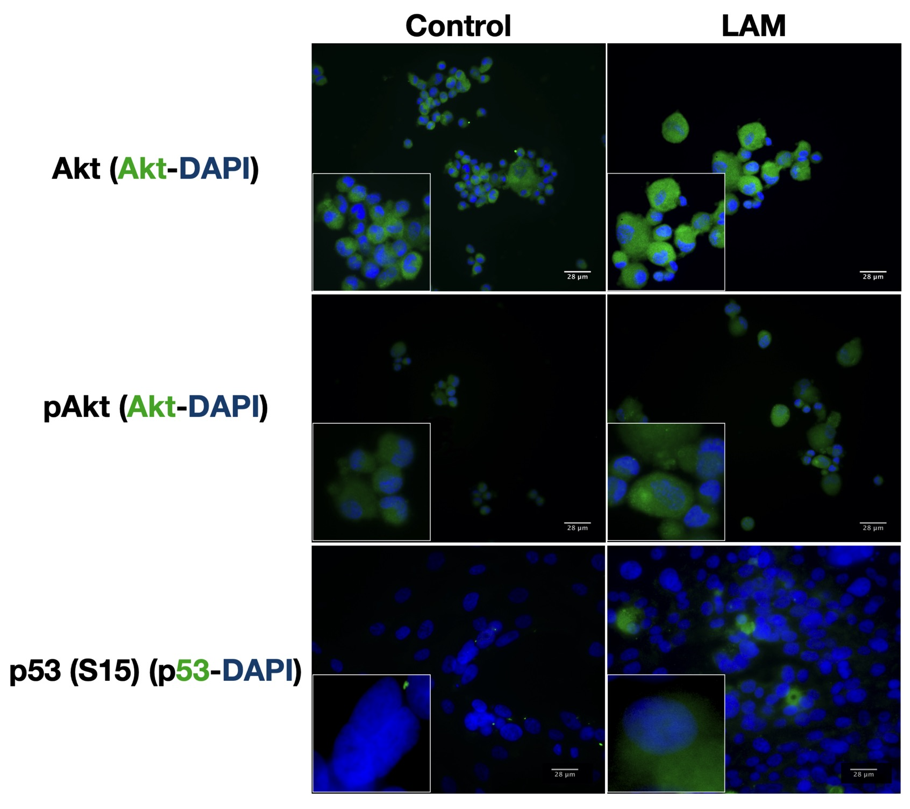
**

**B**

**A**

**S. Figure 2. Phosphorylation cascade. A)** pAKT and p53 (S15) immunofluorescent staining (magnification ×40, size bar 28 μm). **B)** Staining intensity compared to control. Data is presented as staining intensity compared to control ± technical error, significant changes are marked as ★★★ and ★★★★ (P<0.0002 and P<0.0001 respectively).

**S. Figure 3. TRXR activity and ROS production following inducers and inhibitors. A, B)** ROS production following H_2_O_2_ as positive control and glucose starvation as inducer for apoptosis. Fluorescence intensity compared to untreated control or S102 ± technical error [1, 2]. **C, D)** TrxR activity (nmol/min/ml) following inhibition using 750nM Auranofin and after the induction of ROS production [3-5]. Significant changes are marked as ★, ★★, ★★★ and ★★★★ (P<0.05, P<0.001, P<0.0002 and P<0.0001 respectively). TRXR system and Trx and the glutathione (GSH) system composed of NADPH, glutathione reductase, and GSH supported by glutaredoxin are the two electron donor systems that control cellular proliferation, viability, and apoptosis. Mitochondrial damage can lead to increased ROS production and imbalance of TRXR in primary LAM tissue derived cell lines [4]. In response, cells increase their TRXR activity to cope with the increased ROS production and to avoid cell damage and death [5].

**D**

**B**

**A**

**C**

1. Zou P, Chen M, Ji J, Chen W, Chen X, Ying S, et al. Auranofin induces apoptosis by ROS-mediated ER stress and mitochondrial dysfunction and displayed synergistic lethality with piperlongumine in gastric cancer. Oncotarget. 2015.
2. Park N, Chun YJ. Auranofin promotes mitochondrial apoptosis by inducing annexin A5 expression and translocation in human prostate cancer cells. J Toxicol Environ Heal - Part A Curr Issues. 2014.
3. Lopert P, Day BJ, Patel M. Thioredoxin Reductase Deficiency Potentiates Oxidative Stress, Mitochondrial Dysfunction and Cell Death in Dopaminergic Cells. PLoS One. 2012.
4. Abdelwahab EMM, Pal S, Kvell K, Sarosi V, Bai P, Rue R, et al. Mitochondrial dysfunction is a key determinant of the rare disease lymphangioleiomyomatosis and provides a novel therapeutic target. Oncogene. 2019;38:3093–101. doi:10.1038/s41388-018-0625-1.
5. Zorov DB, Juhaszova M, Sollott SJ (2014) Mitochondrial reactive oxygen species (ROS) and ROS-induced ROS release. Physiol. Rev. 94:909–950


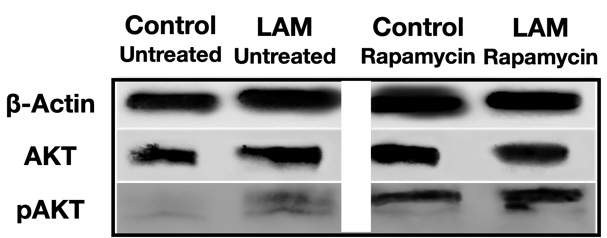
**
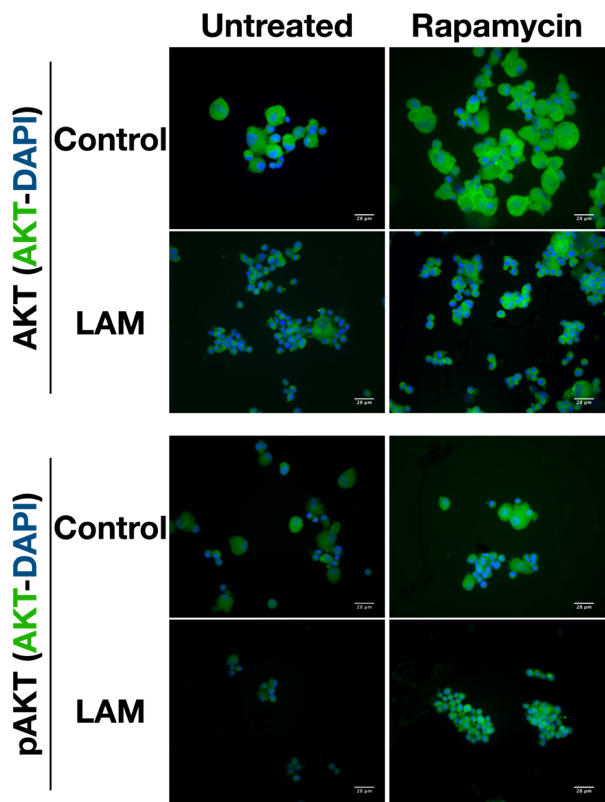
**

**B**

**A**

**S. Figure 4. Phosphorylation cascade following rapamycin treatment. A, B)** AKT and p-AKT western blot and immunofluorescent staining using LAM cell lines (magnification ×40, size bar 28 μm) (AKT, pAKT green, DAPI blue) following rapamycin treatment. Rapamycin induced AKT phosphorylation and decreased the phosphorylation of S6K1 [1-7].

1. Yu J, Astrinidis A, Howard S, Henske EP. Estradiol and tamoxifen stimulate LAM-associated angiomyolipoma cell growth and activate both genomic and nongenomic signaling pathways. American Journal of Physiology - Lung Cellular and Molecular Physiology. 2004;286 4 30-4. doi:10.1152/ajplung.00204.2003.
2. Himes BE, Obraztsova K, Lian L, Shumyatcher M, Rue R, Atochina-Vasserman EN, et al. Rapamycin-independent IGF2 expression in Tsc2-null mouse embryo fibroblasts and human lymphangioleiomyomatosis cells. PLoS One. 2018;13.
3. Siroky BJ, Yin H, Babcock JT, Lu L, Hellmann AR, Dixon BP, et al. Human TSC-associated renal angiomyolipoma cells are hypersensitive to ER stress. Am J Physiol - Ren Physiol. 2012.
4. Hong F, Larrea MD, Doughty C, Kwiatkowski DJ, Squillace R, Slingerland JM. mTOR-Raptor Binds and Activates SGK1 to Regulate p27 Phosphorylation. Mol Cell. 2008.
5. 1. Laplante M, Sabatini DM. mTOR signaling at a glance. J Cell Sci. 2009;122 Pt 20:3589–94. doi:10.1242/jcs.051011.
6. Choo AY, Kim SG, Vander Heiden MG, Mahoney SJ, Vu H, Yoon SO, et al. Glucose Addiction of TSC Null Cells Is Caused by Failed mTORC1-Dependent Balancing of Metabolic Demand with Supply. Mol Cell. 2010.
7. Lee CH, Inoki K, Karbowniczek M, Petroulakis E, Sonenberg N, Henske EP, et al. Constitutive mTOR activation in TSC mutants sensitizes cells to energy starvation and genomic damage via p53. EMBO J. 2007;26:4812–23.

**
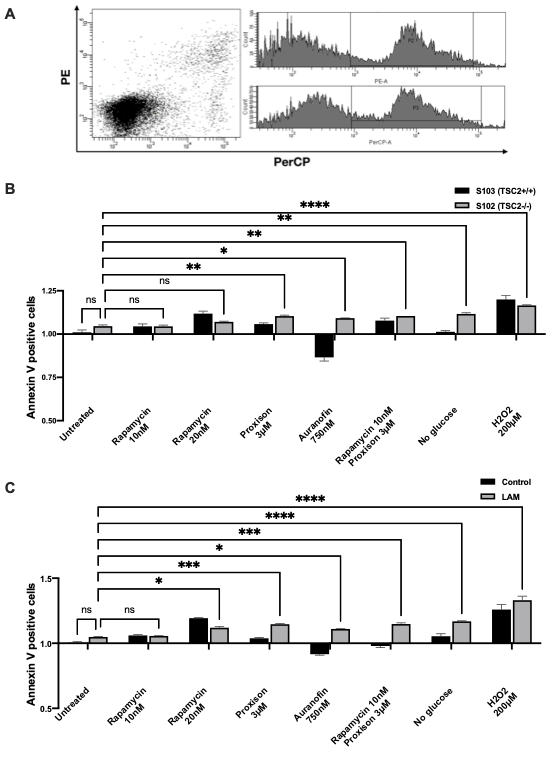
S. Figure 5. Proxison induces apoptosis via ROS production. A)** Flowcytometer analysis of Annexin V-PE and 7-AAD using BD FACSCanto™ II system. **B, C)** Annexin V positive cells compared to untreated control and following all combination of treatments used during the study. Significant changes are marked as ★, ★★, ★★★ and ★★★★ (P<0.05, P<0.001, P<0.0002 and P<0.0001 respectively).

**S. Figure 6. miRNA expression involved in regulation of apoptosis, p53 activation and proliferation.** Results are presented as individual data points LAM n=4 and Controls n=4. Each point is an average of 3 replicates**.**

| **miRNA** | **In TSC-deficit model** | **Target** | **Consequences** | **Ref** |
| --- | --- | --- | --- | --- |
| **199a-3p** | **↓*** | **YAP1** | **Inhibits cell proliferation and induces apoptosis** | **1** |
| **23a-3p** | **↑↑** | **p53** | **Promotes the apoptotic effect of p53** | **2** |
| **128-3p** | **↑** | **p53** | **Positively regulates p53** | **2** |
| **34a-5p** | **↓*** | **p53** | **miR-34a expression is induced by p53 and increases apoptosis** | **4** |
| **215-5p** | **↓↓*** | **Akt/p53** | **Induced by p53 and suppresses cell proliferation through p21** | **3** |
| **378c-d** | **↓↓*** | **SDAD1** | **Suppresses cell proliferation** | **6** |
| **151a-3p** | **↓** | **p53** | **Induces cell proliferation via p53 inhibition** | **7** |
| **101-3p** | **↑** | **p53/cIAPs** | **Regulates cell proliferation independently from p53 and regulates apoptosis via a circuit involving p53** | **8** |
| **103a-3p** | **↑** | **Atg5** | **Induces apoptosis and autophagy via Atg5** | **9** |
| **122-5p** | **↑↑*** | **Akt/p53** | **Inhibits apoptosis via activation of Akt and inhibition of p53** | **5** |
| **1260b** | **↓*** | **Kit signal** | **Promotes cell proliferation and inhibits apoptosis** | **10** |

**** Deregulations affecting apoptosis***

**S. Table 4.** miRNAs involved in cell proliferation and apoptosis regulations.

1. Ren K, Li T, Zhang W, et al (2016) miR-199a-3p inhibits cell proliferation and induces apoptosis by targeting YAP1, suppressing Jagged1-Notch signaling in human hepatocellular carcinoma. J Biomed Sci 23:1–9. https://doi.org/10.1186/s12929-016-0295-7

2. Li J, Aung LHH, Long B, et al (2015) MiR-23a binds to p53 and enhances its association with miR-128 promoter. Sci Rep 5:1–13. https://doi.org/10.1038/srep16422

3. Braun CJ, Zhang X, Savelyeva I, et al (2008) p53-responsive microRNAs 192 and 215 are capable of inducing cell cycle arrest. Cancer Res 68:10094–10104. https://doi.org/10.1158/0008-5472.CAN-08-1569

4. Raver-Shapira N, Marciano E, Meiri E, et al (2007) Transcriptional Activation of miR-34a Contributes to p53-Mediated Apoptosis. Mol Cell 26:731–743. https://doi.org/10.1016/j.molcel.2007.05.017

5. Manfè V, Biskup E, Rosbjerg A, et al (2012) MiR-122 regulates p53/Akt signalling and the chemotherapy-induced apoptosis in cutaneous T-cell lymphoma. PLoS One 7:. https://doi.org/10.1371/journal.pone.0029541

6. Zeng M, Zhu L, Li L, Kang C (2017) miR-378 suppresses the proliferation, migration and invasion of colon cancer cells by inhibiting SDAD1. Cell Mol Biol Lett 22:. https://doi.org/10.1186/s11658-017-0041-5

7. Liu H, Cheng Y, Xu Y, et al (2019) The inhibition of tumor protein p53 by microRNA-151a-3p induced cell proliferation, migration and invasion in nasopharyngeal carcinoma. Biosci Rep 39:. https://doi.org/10.1042/BSR20191357

8. Fujiwara Y, Saito M, Robles AI, et al (2018) A Nucleolar Stress–Specific p53–miR-101 Molecular Circuit Functions as an Intrinsic Tumor-Suppressor Network. EBioMedicine 33:33–48. https://doi.org/10.1016/j.ebiom.2018.06.031

9. Zhang C, Lu J, Wang H, et al (2019) Effects of miR‑103a‑3p on the autophagy and apoptosis of cardiomyocytes by regulating Atg5. Int J Mol Med 43:1951–1960. https://doi.org/10.3892/ijmm.2019.4128

10. Xia Y, Wei K, Yang FM, et al (2019) miR-1260b, mediated by YY1, activates KIT signaling by targeting SOCS6 to regulate cell proliferation and apoptosis in NSCLC. Cell Death Dis 10:1–14. https://doi.org/10.1038/s41419-019-1390-y

**Supplementary material and data**

**Materials and methods**

LAM tissue samples were obtained from lung transplant donors for generation of cell lines, in accordance with the Declaration of Helsinki, approved by the Institutional Review Board at the University of Pennsylvania [1] and provided by the National Disease Research Interchange (NDRI, Philadelphia, PA). LAM patients had given written informed consent and all the collected samples were treated anonymously. Paraffin embedded tissue samples were obtained retrospectively from the Departments of Pathology at Semmelweis University, Budapest, and from the University of Pecs, Pecs, Hungary and the National Koranyi Institute of Pulmonology, Budapest, Hungary. The study was approved by the Medical Research Council of Hungary (54034-4/2018/EKU).

## LAM cell lines, bronchial smooth muscle cells (BSMC), normal human lung fibroblast (NHLF) S102 and S103 cell lines and cell culture conditions

Primary cultures of human LAM cells where established in the Department of Medicine, University of Pennsylvania, Pennsylvania, USA [1]. Briefly, the primary cultures of LAM cells were dissociated from the LAM nodules of transplant patients. Each LAM nodule was used to establish individual cell lines (characterized based on alpha smooth muscle actin (α***-***SMA) expression, mTORC1 activation, HMB45 immunoreactivity, DNA synthesis, and cell migration) [2]. In the current study, four patient-derived individual LAM cell lines were used including LAM-100, LAM-111C, LAM-D9065 and LAM-HUP. As controls, primary cultures of normal, human bronchial smooth muscle cells (BSMC) and normal human lung fibroblasts (NHLF), were purchased from Lonza (Basel, Switzerland). Normal, BSMC and LAM cells were cultured at 37°C, 5% CO_2_ in SMC Growth Medium (insulin, hFGF, GA, FBS and hEGF) (Lonza, Basel, Switzerland). The 621-101 cells were derived from an angiomyolipoma and carried bi-allelic inactivation of the TSC2 gene [3, 4]. The 621-102 cell line was generated by introduction of E6/E7 (pLXSN 16E6E7-neo) and human telomerase (pLXSN hTERT-hyg) into a primary culture of TSC2 null human angiomyolipoma cells [5]. 621-103 was generated by stable transfection of TRI102 with wild-type TSC2 (pcDNA3.1 TSC2-zeo) into 621-101 cells [3].

## Haematoxylin eosin staining

5 µm thick tissue sections were stained in Mayer’s haematoxylin solution (Sigma-Aldrich, St. Louis, USA) for 10 min, washed, then differentiated with 0.25% acetic acid and in eosin solution. Sections were mounted using Vectashield mounting medium (Vector Laboratories, Burlingame, USA). Images were taken using Nikon Eclipse Ti-U inverted microscope.

## Immunofluorescent staining

Normal, BSMC, NHLF, LAM, S103 and S102 cells were cultured for 3 days using Falcon™ chambered cell culture slides (Thermo Fisher Scientific, Waltham, USA) or Cytospins were made from cell suspension. Cell cultures were then fixed in 4% formaldehyde and permeabilized using PBS containing 0.1% Triton-X and 5% BSA.

**S. Table 1.** Antibodies used in immunofluorescent staining, immunohistochemistry and western blotting

| Anti-alpha -Smooth Muscle Actin | MAB1420 (1∶100) |
| --- | --- |
| Anti-mTOR Antibody | ab25880 (1:100) |
| Anti-p70 S6 kinase Antibody | Ab32529 (1:100) |
| anti-HMB-45 | Ab787 (1:100) |
| Anti-human Akt | Cell Signaling 9272 (1:1000) |
| Anti-human pAkt | Cell Signaling 9275 (1:0000) |
| Anti-human P53 | ab1431 (1:200) |
| Anti-beta actin | Cell Signaling 4970 (1:0000) |
| Anti-mouse Alexa 488 | A28175 (1:200) |
| Anti-rabbit Alexa 647 | A27040 (1:200) |
| Anti-rabbit Alexa 488 | A11034 (1:200) |
| Anti-mouse Alexa 647 | A32728 (1:200) |
| Anti-mouse HRP | 170-6516 (1:2000) |
| Anti-rabbit HRP | 170-6515 (1:2000) |
| Anti-rabbit HRP | P0448 (1:200) |

Nuclei were counter stained with DAPI. Images were acquired using an Olympus IX-81 (OLYMPUS Corporation, Tokyo, Japan) both light and fluorescence microscope.

**Immunohistochemistry**

5 µm thick tissue sections were stained using immunohistochemistry. First, the slides were rinsed in heated xylene and were washed with a descending series of alcohol to remove paraffin. After deparaffination the slides were rehydrated in distilled water and antigen retrieval was performed by heating the slides in Target Retrieval Solution (pH 6, DAKO, Produktionsvej, Denmark) at 97°C for 20–30 min. Subsequently slides were washed in dH_2_O and endogenous peroxidase activity was blocked with 3% H_2_O_2_ containing TBS (pH 7.4) for 15 min. Then slides were washed three times with TBS containing Tween (0.05%, pH 7.4). Pre-blocking was carried out with 3% BSA in TBS for 20 min before overnight incubation with anti- Melanoma gp100 antibody (HMB-45) (1:100, HMB-45 mouse monoclonal antibody clone: Ab787, ABcam) primary antibody at 4°C. Following incubation slides were washed with TBS for three times then incubated with peroxidase conjugated secondary antibody (1:100, Polyclonal Goat Anti-Rabbit IgG, DAKO) for 90 min. Antibody labelling was visualized with the help of liquid DAB Substrate Chromogen System (DAKO). For nuclear counterstaining, haematoxylin staining was performed. Finally, slides were mounted with Faramount Aqueous Mounting Medium (DAKO, Produktionsvej, Denmark). Histological evaluation was performed with the help of Panoramic MIDI digital slide scanner (3DHistech, Budapest, Hungary). Image analysis was performed using ImageJ software with IHC toolbox plug-in.

### **Rapamycin, Proxison, Auranofin and H_2_O_2_ treatment**

BSMC, NHLF, LAM, S103 and S102 cell cultures were treated with mono rapamycin or Proxison treatments and in combined treatments using the following concentrations: 10 or 20 nM Rapamycin catalogue: tlrl-rap (InvivoGen, San Diego, USA) and 3 µM Proxison (Antoxis Ltd, Aberdeen, UK) for 24h at 37°C, 5% CO_2_. Auranofin and H_2_O_2_ were used as positive controls for TrxR activity, ROS production and apoptosis analysis (Annexin V) using the following concentrations: 750 nM Auranofin (Thermo Scientific, Waltham, MA) and 200 µM H_2_O_2_ (Thermo Scientific, Waltham, MA).

**Western blot**

Cells were lysed in ice-cold RIPA buffer (Sigma-Aldrich, St. Louis, USA) supplemented with protease inhibitors (Roche Diagnostics, Mannheim, Germany) for 30 min on ice and centrifuged at 16,000 × g for 20 min at 4° C. The supernatant was then used as the cell lysate. The protein content of each cell lysate was assessed using a Qubit protein assay kit (Thermo Scientific, Waltham, MA). 30 µg of total protein was loaded onto Mini Protean gel (Bio-Rad, California, USA), then electrophoresis was followed by overnight blotting onto a nitrocellulose membrane using 10 mA current. The blots then were blocked in 5% non-fat skimmed milk blocking solution (Bio-Rad, California, USA) in TBS-T for 1 h and incubated with primary antibodies diluted 1:1000 in 2.5% non-fat skimmed milk powder in TBS-T overnight at 4° C. After washing with TBS-T, the blots were incubated with rabbit anti-goat/HRP diluted in 2.5% non-fat skimmed milk powder in TBS-T for 1 h at room temperature. The immunoreaction was developed with a chemiluminescence HRP substrate and recorded with ImageQuant LAS-4000 imager (GE Healthcare Life Sciences, USA).

## RNA isolation

Total RNA was extracted from normal BSMC, NHLF and LAM cell cultures with MN NucleoSpin RNA isolation kit according to the manufacturer’s protocol (Macherey-Nagel, Düren, Germany). The concentration of RNA samples was measured using NanoDrop (Thermo Fisher Scientific, Waltham, USA). Total RNA from human lung tissues were obtained using TRIzol reagent (Invitrogen, Thermo Fisher Scientific, Waltham, USA). RNA (1 µg) was digested with DNase (Sigma-Aldrich, St. Louis, USA) to eliminate any DNA contamination. cDNA was synthesized with high capacity RNA to cDNA kit (Thermo Fisher Scientific, Waltham, USA). Reverse transcription was performed with random hexamer primers.

## Quantitative qRT-PCR

qRT-PCR was performed using SensiFAST SYBR Green reagent (BioLine, London, UK) in an ABI StepOnePlus system. Gene expressions using sequence specific primers (Table 2) were analysed with StepOne software and normalized to beta-actin. Changes in gene expression were calculated according to the 2^-ddCt^ method.

**S. Table 2.** Primer sequences

| **Gene name** | **Forward primer** | **Reverse primer** |
| --- | --- | --- |
| **β-actin** | GCGCGGCTACAGCTTCA | CTTAATGTCACGCACGATTTCC |
| **TSC1** | CCGTGGCCCTATGCTTGTAA | CGGCTTTGCCCACATATTCG |
| **TSC2** | CCTTGGACGGTATTGCCTGT | GCCTGCTTCTGTGTACCACT |

**Quantstudio 12k flex**

cDNA was prepared using TaqMan miRNA reverse transcriptase kit and Megaplex RT primers Pool A and B (Thermo Fisher Scientific, Waltham, USA) according to manufacturers’ protocol using 350ng-1000ng of total RNA as starting material. miRNA expression levels were performed using open array miRNA card Pool A and B and Quantstudio 12k flex (Thermo Fisher Scientific, Waltham, USA).

**Protein arrays**

Cell lysates of 1 x 107 103 cells/ml were assessed using a Human phosphor-kinases and apoptosis Array Kits (R&D Systems, Minneapolis, USA). Protein concentration was determined using a fluorescent protein assay (Qubit Protein, Thermo Fisher Scientific, Waltham, USA). Briefly, the Detection Antibody Cocktail was mixed with each sample and incubated with the membrane at 4°C overnight, then with Streptavidin-HRP at room temperature finally with the Chemiluminescent-Reagent Mix. Images were captured using LAS-4000 (GE Healthcare Bio-Sciences AB Uppsala, Sweden), and intensity was determined using ImageJ (https://imagej.nih.gov/ij/) and normalized to the reference spots.

**Cell Viability Assay**

CellTiter-Glo Luminescent Cell Viability Assay Kit (Promega Corp., Madison, WI, USA) was used to evaluate cell viability after drug treatment. Cells were seeded into 96-well plates, after 24 h incubation and were treated with mono rapamycin or RA or Proxison and their combinations. After incubation for 24 h at 37°C, 100 μl of CellTiter-Glo reagent were added and luminescence measured with EnSpire® Multimode Plate Reader (PerkinElmer, Waltham, Massachusetts, USA). Pierce™ BCA Protein Assay Kit (Thermo Fisher Scientific, Waltham, USA) was used to measure protein content and results are presented as the fold change vs. control.

**Measurement of intracellular ROS levels**

Reactive oxygen species were measured using green Fluorometric Intracellular Ros Kit (Sigma-Aldrich, St. Louis, USA). Cells were grown in 96-well plates and treated then ROS levels were assessed using a detection kit following the manufacturer’s instructions and results were measure at Ex/Em = 490/525 nm with EnSpire® Multimode Plate Reader (PerkinElmer, Waltham, Massachusetts, USA). Pierce™ BCA Protein Assay Kit (Thermo Fisher Scientific, Waltham, USA) was used and results are presented as the fold change vs. control.

**Measurement of Thioredoxin Reductase Activity**

Thioredoxin reductase (TrxR) activity was measured in cell homogenates using a thioredoxin reductase assay kit according to the manufacturer's instructions (Abcam, MA, USA, ab83463). Briefly, cells were cultured in 96-well plates and incubated with treatment for 24 h at 37°C. Cells were collected and homogenized on ice by adding 100 μL cold assay buffer. Supernatants were collected by centrifugation at 10,000 ×g for 15 min, and TrxR activity was measured according to the kit's instructions.

**Flow cytometry**

50,000 cells were collected from each cell cultures and incubated with Annexin V-PE and 7-AAD (BioLegend, San Diego, CA, USA) for 15 mins at 37^o^C. Using BD FACSCanto™ II system (three lasers: blue 488-nm, air-cooled, 20-mW solid state, red 633-nm, 17-mW HeNe, and violet 405-nm, 30-mW solid state). Labeled cells were analyzed using FACS Canto II flow cytometer (BD Immunocytometry Systems, Erembodegen, Belgium) with BD FACS DIVA software V6 and data were analyzed by FCS Express V3 software.

## Statistical analysis

Unless otherwise noted, statistical analysis was performed with SPSS version 20 software. S102 and S103 data are presented as mean ± technical error of three replicates and statistical analysis was performed using the t-test. LAM primary samples and their controls (BSMC n=4 and NHLF n=4) data are presented as mean ± standard error of mean (SEM), and statistical analysis was performed using the one-way ANOVA. p<0.05 was considered as significant.

1. Goncharova EA, Goncharov DA, Eszterhas A, et al (2002) Tuberin regulates p70 S6 kinase activation and ribosomal protein S6 phosphorylation: A role for the TSC2 tumor suppressor gene in pulmonary lymphangioleiomyomatosis (LAM). J Biol Chem 277:30958–30967. https://doi.org/10.1074/jbc.M202678200

2. Goncharova EA, Goncharov DA, Lim PN, et al (2006) Modulation of cell migration and invasiveness by tumor suppressor TSC2 in lymphangioleiomyomatosis. Am J Respir Cell Mol Biol 34:473–480. https://doi.org/10.1165/rcmb.2005-0374OC

3. Yu J, Astrinidis A, Howard S, Henske EP (2004) Estradiol and tamoxifen stimulate LAM-associated angiomyolipoma cell growth and activate both genomic and nongenomic signaling pathways. Am. J. Physiol. - Lung Cell. Mol. Physiol. 286

4. Carsillo T, Astrinidis A, Henske EP (2000) Mutations in the tuberous sclerosis complex gene TSC2 are a cause of sporadic pulmonary lymphangioleiomyomatosis. Proc Natl Acad Sci U S A 97:6085–6090. https://doi.org/10.1073/pnas.97.11.6085

5. Furukawa T, Duguid WP, Rosenberg L, et al (1996) Long-term culture and immortalization of epithelial cells from normal adult human pancreatic ducts transfected by the E6E7 gene of human papilloma virus 16. Am J Pathol 148:1763–1770

S. Table 3. GeneMANIA gene expression analysis.

*
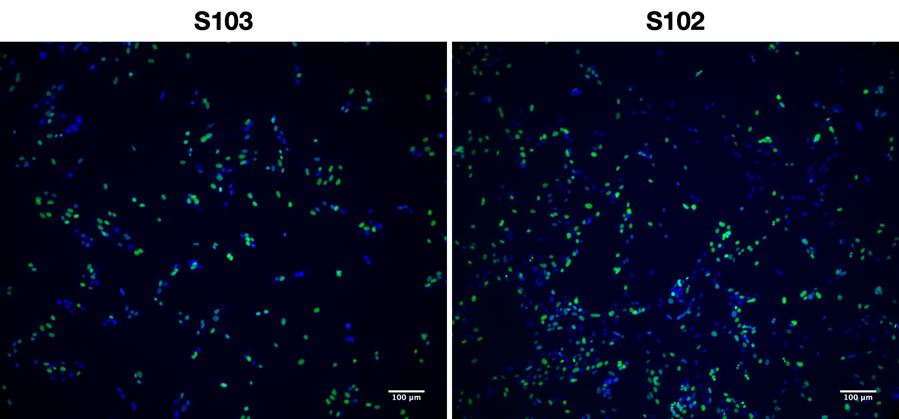
*

**S. Figure 1. Proliferation capacity of S102 compared to S103 using BrdU assay (BrdU green, DAPI blue, size-bar 100 μm)**.

**
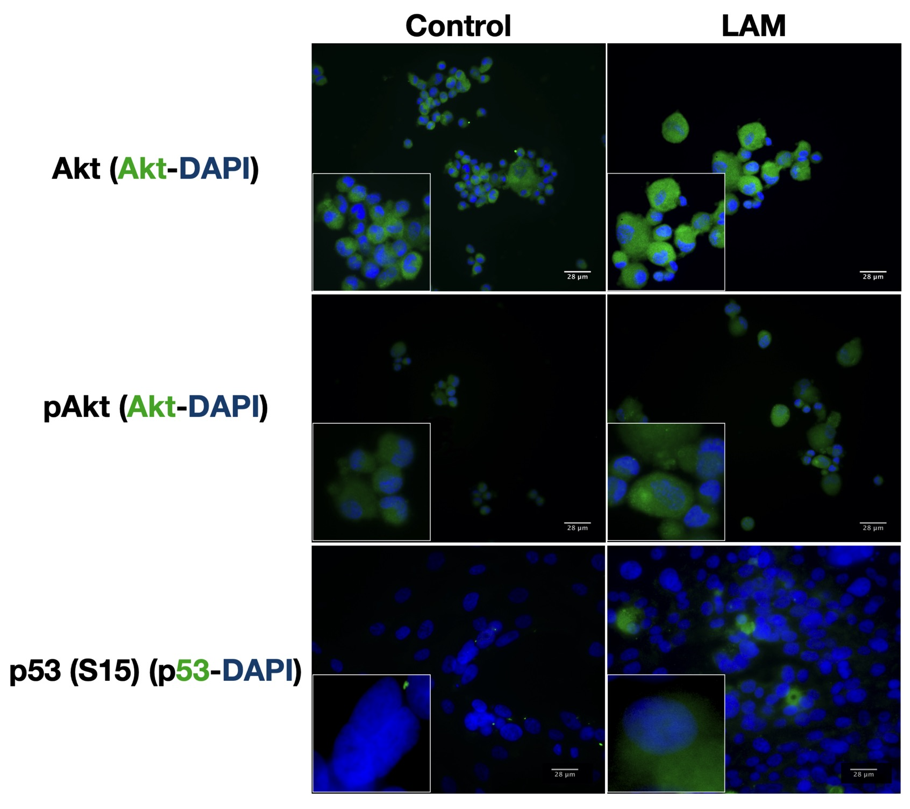
**

**B**

**A**

**S. Figure 2. Phosphorylation cascade. A)** pAKT and p53 (S15) immunofluorescent staining (magnification ×40, size bar 28 μm). **B)** Staining intensity compared to control. Data is presented as staining intensity compared to control ± technical error, significant changes are marked as ★★★ and ★★★★ (P<0.0002 and P<0.0001 respectively).

**S. Figure 3. TRXR activity and ROS production following inducers and inhibitors. A, B)** ROS production following H_2_O_2_ as positive control and glucose starvation as inducer for apoptosis. Fluorescence intensity compared to untreated control or S102 ± technical error [1, 2]. **C, D)** TrxR activity (nmol/min/ml) following inhibition using 750nM Auranofin and after the induction of ROS production [3-5]. Significant changes are marked as ★, ★★, ★★★ and ★★★★ (P<0.05, P<0.001, P<0.0002 and P<0.0001 respectively). TRXR system and Trx and the glutathione (GSH) system composed of NADPH, glutathione reductase, and GSH supported by glutaredoxin are the two electron donor systems that control cellular proliferation, viability, and apoptosis. Mitochondrial damage can lead to increased ROS production and imbalance of TRXR in primary LAM tissue derived cell lines [4]. In response, cells increase their TRXR activity to cope with the increased ROS production and to avoid cell damage and death [5].

**D**

**B**

**A**

**C**

1. Zou P, Chen M, Ji J, Chen W, Chen X, Ying S, et al. Auranofin induces apoptosis by ROS-mediated ER stress and mitochondrial dysfunction and displayed synergistic lethality with piperlongumine in gastric cancer. Oncotarget. 2015.
2. Park N, Chun YJ. Auranofin promotes mitochondrial apoptosis by inducing annexin A5 expression and translocation in human prostate cancer cells. J Toxicol Environ Heal - Part A Curr Issues. 2014.
3. Lopert P, Day BJ, Patel M. Thioredoxin Reductase Deficiency Potentiates Oxidative Stress, Mitochondrial Dysfunction and Cell Death in Dopaminergic Cells. PLoS One. 2012.
4. Abdelwahab EMM, Pal S, Kvell K, Sarosi V, Bai P, Rue R, et al. Mitochondrial dysfunction is a key determinant of the rare disease lymphangioleiomyomatosis and provides a novel therapeutic target. Oncogene. 2019;38:3093–101. doi:10.1038/s41388-018-0625-1.
5. Zorov DB, Juhaszova M, Sollott SJ (2014) Mitochondrial reactive oxygen species (ROS) and ROS-induced ROS release. Physiol. Rev. 94:909–950


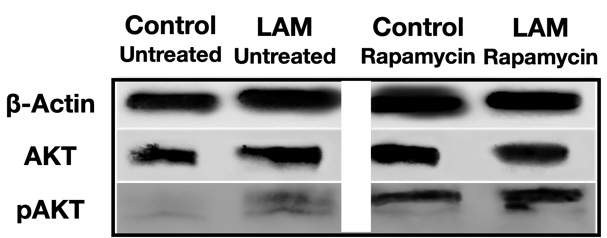
**
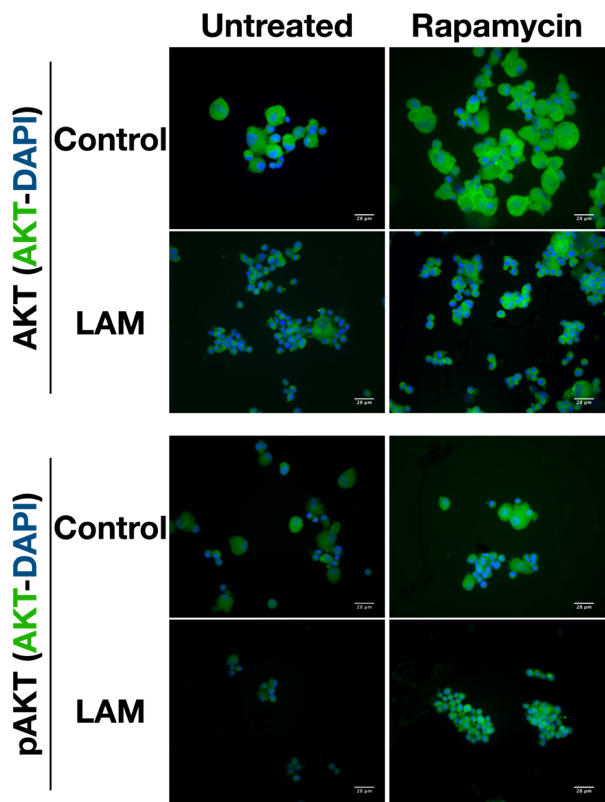
**

**B**

**A**

**S. Figure 4. Phosphorylation cascade following rapamycin treatment. A, B)** AKT and p-AKT western blot and immunofluorescent staining using LAM cell lines (magnification ×40, size bar 28 μm) (AKT, pAKT green, DAPI blue) following rapamycin treatment. Rapamycin induced AKT phosphorylation and decreased the phosphorylation of S6K1 [1-7].

1. Yu J, Astrinidis A, Howard S, Henske EP. Estradiol and tamoxifen stimulate LAM-associated angiomyolipoma cell growth and activate both genomic and nongenomic signaling pathways. American Journal of Physiology - Lung Cellular and Molecular Physiology. 2004;286 4 30-4. doi:10.1152/ajplung.00204.2003.
2. Himes BE, Obraztsova K, Lian L, Shumyatcher M, Rue R, Atochina-Vasserman EN, et al. Rapamycin-independent IGF2 expression in Tsc2-null mouse embryo fibroblasts and human lymphangioleiomyomatosis cells. PLoS One. 2018;13.
3. Siroky BJ, Yin H, Babcock JT, Lu L, Hellmann AR, Dixon BP, et al. Human TSC-associated renal angiomyolipoma cells are hypersensitive to ER stress. Am J Physiol - Ren Physiol. 2012.
4. Hong F, Larrea MD, Doughty C, Kwiatkowski DJ, Squillace R, Slingerland JM. mTOR-Raptor Binds and Activates SGK1 to Regulate p27 Phosphorylation. Mol Cell. 2008.
5. 1. Laplante M, Sabatini DM. mTOR signaling at a glance. J Cell Sci. 2009;122 Pt 20:3589–94. doi:10.1242/jcs.051011.
6. Choo AY, Kim SG, Vander Heiden MG, Mahoney SJ, Vu H, Yoon SO, et al. Glucose Addiction of TSC Null Cells Is Caused by Failed mTORC1-Dependent Balancing of Metabolic Demand with Supply. Mol Cell. 2010.
7. Lee CH, Inoki K, Karbowniczek M, Petroulakis E, Sonenberg N, Henske EP, et al. Constitutive mTOR activation in TSC mutants sensitizes cells to energy starvation and genomic damage via p53. EMBO J. 2007;26:4812–23.

**
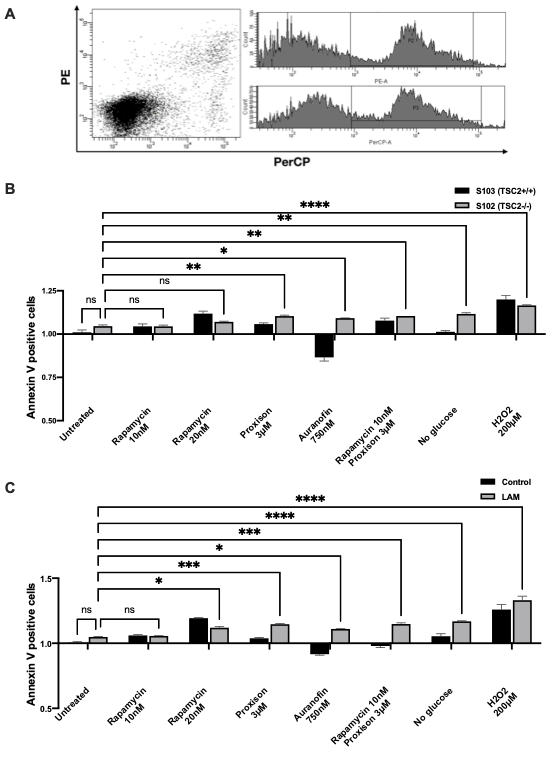
S. Figure 5. Proxison induces apoptosis via ROS production. A)** Flowcytometer analysis of Annexin V-PE and 7-AAD using BD FACSCanto™ II system. **B, C)** Annexin V positive cells compared to untreated control and following all combination of treatments used during the study. Significant changes are marked as ★, ★★, ★★★ and ★★★★ (P<0.05, P<0.001, P<0.0002 and P<0.0001 respectively).

**S. Figure 6. miRNA expression involved in regulation of apoptosis, p53 activation and proliferation.** Results are presented as individual data points LAM n=4 and Controls n=4. Each point is an average of 3 replicates**.**

| **miRNA** | **In TSC-deficit model** | **Target** | **Consequences** | **Ref** |
| --- | --- | --- | --- | --- |
| **199a-3p** | **↓*** | **YAP1** | **Inhibits cell proliferation and induces apoptosis** | **1** |
| **23a-3p** | **↑↑** | **p53** | **Promotes the apoptotic effect of p53** | **2** |
| **128-3p** | **↑** | **p53** | **Positively regulates p53** | **2** |
| **34a-5p** | **↓*** | **p53** | **miR-34a expression is induced by p53 and increases apoptosis** | **4** |
| **215-5p** | **↓↓*** | **Akt/p53** | **Induced by p53 and suppresses cell proliferation through p21** | **3** |
| **378c-d** | **↓↓*** | **SDAD1** | **Suppresses cell proliferation** | **6** |
| **151a-3p** | **↓** | **p53** | **Induces cell proliferation via p53 inhibition** | **7** |
| **101-3p** | **↑** | **p53/cIAPs** | **Regulates cell proliferation independently from p53 and regulates apoptosis via a circuit involving p53** | **8** |
| **103a-3p** | **↑** | **Atg5** | **Induces apoptosis and autophagy via Atg5** | **9** |
| **122-5p** | **↑↑*** | **Akt/p53** | **Inhibits apoptosis via activation of Akt and inhibition of p53** | **5** |
| **1260b** | **↓*** | **Kit signal** | **Promotes cell proliferation and inhibits apoptosis** | **10** |

**** Deregulations affecting apoptosis***

**S. Table 4.** miRNAs involved in cell proliferation and apoptosis regulations.

1. Ren K, Li T, Zhang W, et al (2016) miR-199a-3p inhibits cell proliferation and induces apoptosis by targeting YAP1, suppressing Jagged1-Notch signaling in human hepatocellular carcinoma. J Biomed Sci 23:1–9. https://doi.org/10.1186/s12929-016-0295-7

2. Li J, Aung LHH, Long B, et al (2015) MiR-23a binds to p53 and enhances its association with miR-128 promoter. Sci Rep 5:1–13. https://doi.org/10.1038/srep16422

3. Braun CJ, Zhang X, Savelyeva I, et al (2008) p53-responsive microRNAs 192 and 215 are capable of inducing cell cycle arrest. Cancer Res 68:10094–10104. https://doi.org/10.1158/0008-5472.CAN-08-1569

4. Raver-Shapira N, Marciano E, Meiri E, et al (2007) Transcriptional Activation of miR-34a Contributes to p53-Mediated Apoptosis. Mol Cell 26:731–743. https://doi.org/10.1016/j.molcel.2007.05.017

5. Manfè V, Biskup E, Rosbjerg A, et al (2012) MiR-122 regulates p53/Akt signalling and the chemotherapy-induced apoptosis in cutaneous T-cell lymphoma. PLoS One 7:. https://doi.org/10.1371/journal.pone.0029541

6. Zeng M, Zhu L, Li L, Kang C (2017) miR-378 suppresses the proliferation, migration and invasion of colon cancer cells by inhibiting SDAD1. Cell Mol Biol Lett 22:. https://doi.org/10.1186/s11658-017-0041-5

7. Liu H, Cheng Y, Xu Y, et al (2019) The inhibition of tumor protein p53 by microRNA-151a-3p induced cell proliferation, migration and invasion in nasopharyngeal carcinoma. Biosci Rep 39:. https://doi.org/10.1042/BSR20191357

8. Fujiwara Y, Saito M, Robles AI, et al (2018) A Nucleolar Stress–Specific p53–miR-101 Molecular Circuit Functions as an Intrinsic Tumor-Suppressor Network. EBioMedicine 33:33–48. https://doi.org/10.1016/j.ebiom.2018.06.031

9. Zhang C, Lu J, Wang H, et al (2019) Effects of miR‑103a‑3p on the autophagy and apoptosis of cardiomyocytes by regulating Atg5. Int J Mol Med 43:1951–1960. https://doi.org/10.3892/ijmm.2019.4128

10. Xia Y, Wei K, Yang FM, et al (2019) miR-1260b, mediated by YY1, activates KIT signaling by targeting SOCS6 to regulate cell proliferation and apoptosis in NSCLC. Cell Death Dis 10:1–14. https://doi.org/10.1038/s41419-019-1390-y
